# Ocular Injury Caused by the Asian Weaver Ant

**DOI:** 10.7759/cureus.92569

**Published:** 2025-09-17

**Authors:** Tan Aik Kah

**Affiliations:** 1 Ophthalmology, Normah Medical Specialist Centre, Kuching, MYS

**Keywords:** asian weaver ant, chemical injury, formic acid, ocular injury, trauma

## Abstract

This report presents a rare case of ocular injury caused by an Asian weaver ant (*Oecophylla smaragdina*) in a 29-year-old male. The patient sustained a direct ant bite to the conjunctiva, with the ant’s mandibles deeply embedded in the tissue. The unique clinical presentation and subsequent surgical removal of the ant *en bloc* with surrounding conjunctival tissue are described. Postoperative management with topical antibiotics, topical steroids, and oral antibiotics resulted in complete recovery without visual impairment. This case underscores the importance of meticulous foreign body removal in ocular trauma and adds to the limited literature on ant-related eye injuries from this species.

## Introduction

The Asian weaver ant (*Oecophylla smaragdina*) is known for its painful bite and secretion of formic acid, which can cause skin irritation and localized inflammation; acid-related ocular surface damage has also been described in other ant species [1]. These ants are commonly found in trees and foliage, and accidental exposure may occur when they fall onto clothing or become trapped during activities such as helmet removal. Ant-related ocular injuries are rarely reported, and cases of significant ocular injury from this species are exceedingly uncommon [2-5]. The objective of this report is to illustrate the recognition and management of ant-related conjunctival foreign bodies, emphasizing the need for surgical excision when mandibles are firmly embedded. In suspected cases with potential chemical involvement, immediate priorities include ocular surface irrigation and pH assessment to limit tissue damage. This case underscores the importance of careful recognition and timely treatment to ensure full recovery.

## Case presentation

A 29-year-old man presented with severe pain (8/10) and a foreign body sensation in his left eye, two hours after sustaining an injury. He reported that while wearing a safety helmet, ants had crawled onto his head; in removing the helmet, one ant entered his eye. His medical and surgical history was unremarkable, and tetanus vaccination was up to date. No history of allergy or angioedema was reported.

**Figure 1:**
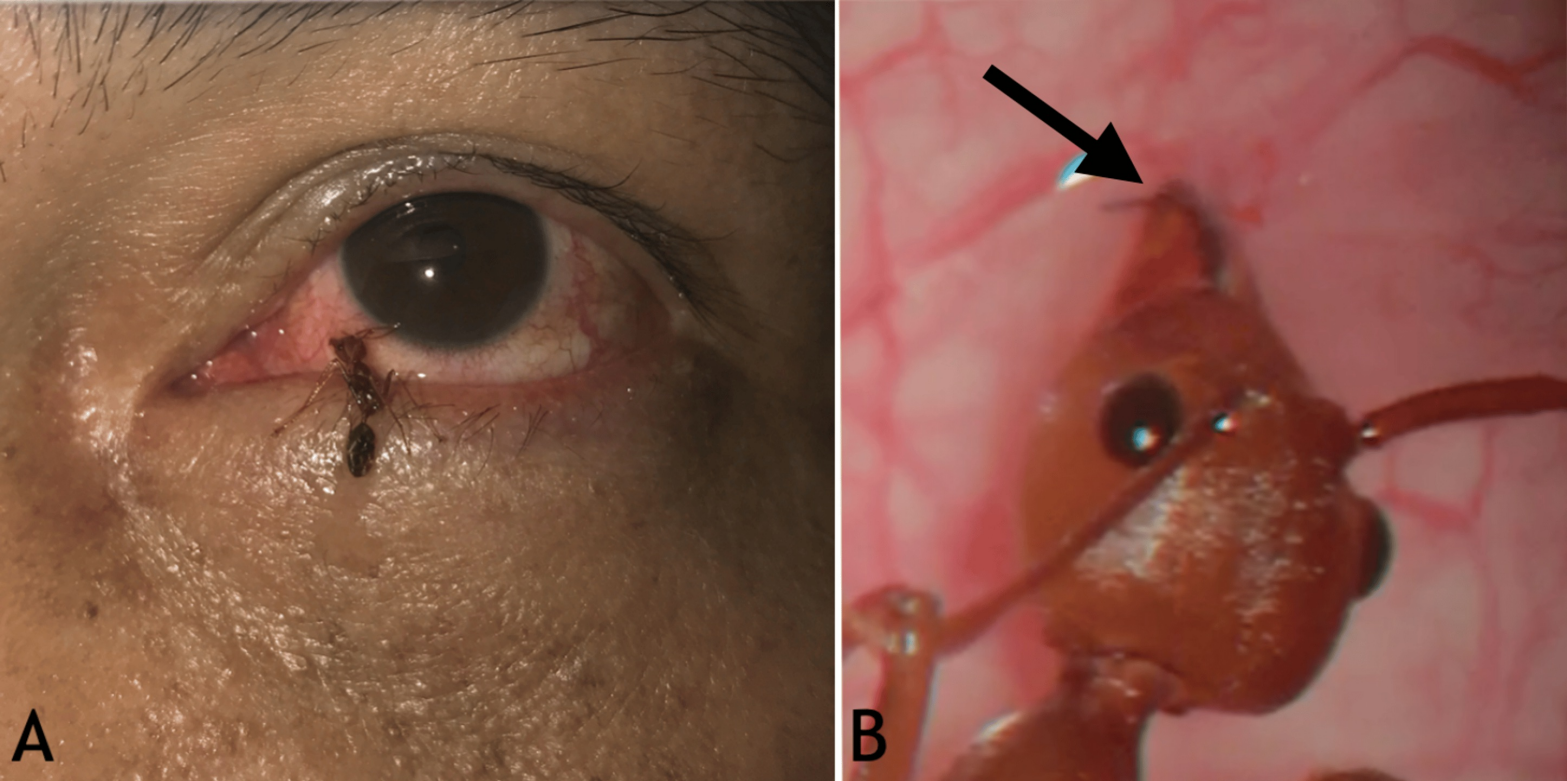
(A) Dead Oecophylla smaragdina (Asian weaver ant) clinging to the patient’s left eye (×1 magnification). (B) Slit-lamp biomicroscopy image (×5 magnification) showing the ant’s head with mandibles deeply embedded in the inferior bulbar conjunctiva (arrow).

On examination, visual acuity was 6/6 in both eyes, and ocular motility was normal. External examination revealed a dead Asian weaver ant attached to the surface of the left eye. Slit-lamp biomicroscopy demonstrated the ant’s head and mandibles deeply embedded in the inferior bulbar conjunctiva, with surrounding conjunctival injection (Figure 1).

Ocular surface pH was not assessed at presentation. Fluorescein staining showed no corneal epithelial defect. Intraocular pressure was 16 mmHg (right) and 14 mmHg (left). The cornea, anterior chamber, iris, and lens were normal, and fundus examination was unremarkable.

**Figure 2:**
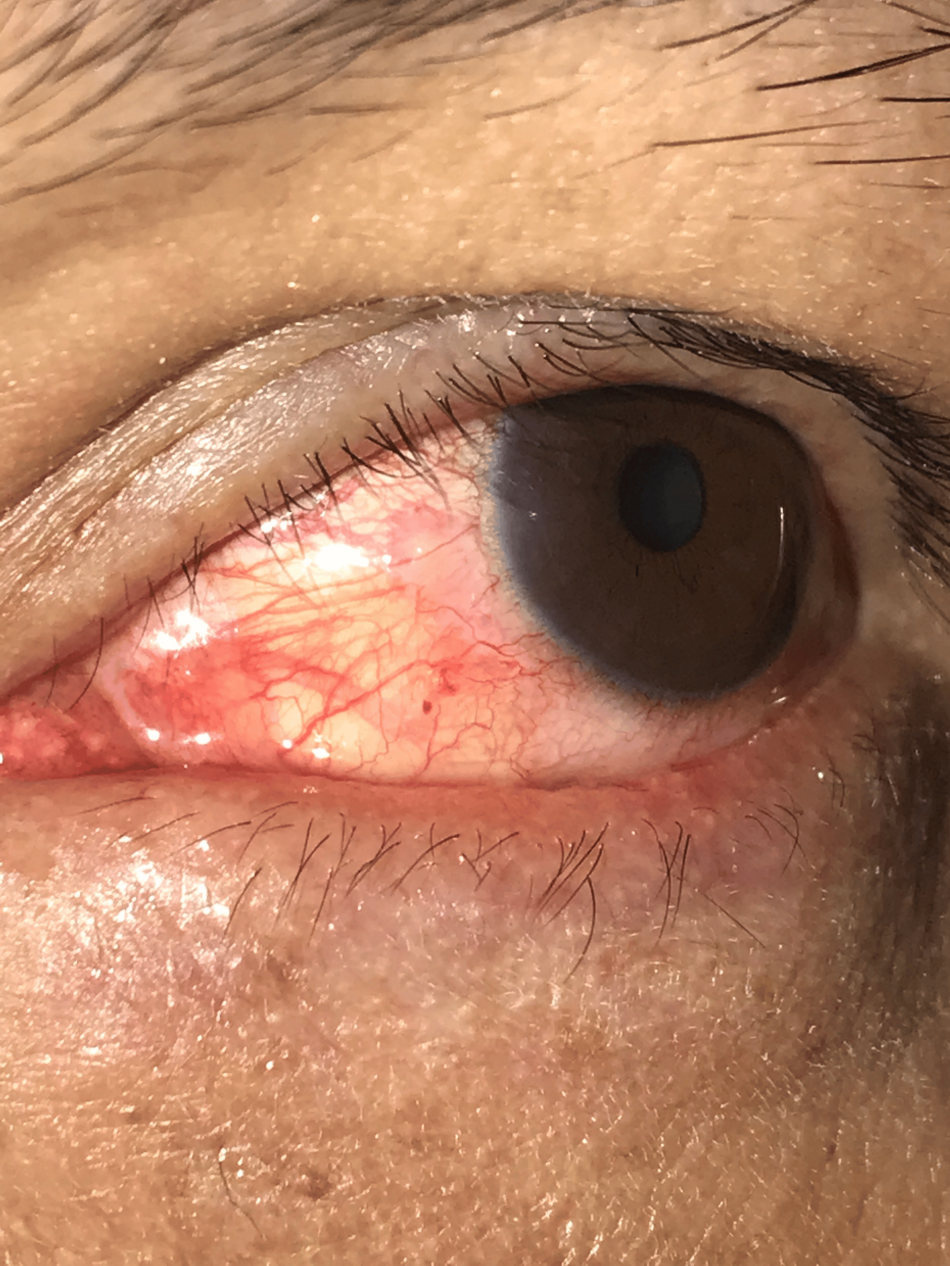
Post-removal slit-lamp image of the left eye showing a clean conjunctival wound without residual foreign body (×2 magnification).

Given the deep embedding, simple extraction was avoided to reduce the risk of retained fragments. Under topical proparacaine hydrochloride 0.5% and antisepsis with 5% povidone-iodine, the ant’s body was grasped with fine-toothed forceps. The conjunctival tissue adherent to the ant’s mandibles was excised with Vannas scissors, allowing en bloc removal (Figure 2).

The conjunctival sac was irrigated thoroughly with approximately 500 mL of sterile water over 5 minutes to eliminate residual debris and possible formic acid exposure. As the excised conjunctival tissue was minimal and not preserved, microbiology/histopathology was not performed.

The patient was prescribed topical Maxitrol® (neomycin, polymyxin B, and dexamethasone) one drop every 4 hours for one week. Oral amoxicillin-clavulanate 625 mg twice daily was given for 5 days, considering the contaminated foreign body and soft tissue involvement (Table 1).

**Table 1:** Summary of prescribed medications.

Agent	Dose	Frequency	Duration
Topical Maxitrol® (neomycin, polymyxin B, and dexamethasone)	1 drop, left eye	Every 4 hours	1 week
Oral amoxicillin-clavulanate	625 mg	Twice daily	5 days

At one-week follow-up, the patient reported complete resolution of symptoms. The conjunctival wound had healed without signs of infection or inflammation, and visual acuity remained 6/6.

## Discussion

Previous reports of insect-related ocular foreign bodies are rare but provide useful context. Lim CH described a case of a conjunctival foreign body due to an ant bite that was managed with simple removal under slit-lamp guidance [3]. Chaurewar AK et al. reported ocular injury caused by fire ants in an unconscious patient, highlighting the risk of prolonged exposure and surface damage [4]. Paz A and Potasman I noted acute ocular pain following exposure to secretions from the electrical ant *Wasmannia auropunctata*, presenting more as a chemical irritation than a mechanical embedding [5]. Compared with these cases, the present report is unique in demonstrating deep mandibular anchoring of the Asian weaver ant into the conjunctiva, necessitating en bloc excision rather than standard extraction or irrigation. The Asian weaver ant is an aggressive species with strong mandibles used for nest construction and prey capture. In this case, the mandibles acted as anchors, firmly embedding the ant into the conjunctiva. Unlike typical superficial ocular foreign bodies that can be irrigated or dislodged, this injury required surgical excision for complete removal.

Although *O. smaragdina* is known to release formic acid as a defensive mechanism, there was no direct evidence of chemical injury in this case (no epithelial defect, corneal haze, or abnormal pH findings). Therefore, the possibility of chemical involvement remains speculative, and the management strategy primarily addressed the mechanical embedding of the mandibles, with irrigation performed as a precaution.

The treatment strategy of en bloc surgical removal combined with topical antibiotics, topical corticosteroids, and systemic antibiotics proved effective. The steroid component controlled inflammation, while antibiotics minimized the risk of secondary infection from a contaminated foreign body. Based on experience and published cases, a practical checklist for clinicians managing ant-related conjunctival foreign bodies is proposed (Table 2).

**Table 2:** Practical checklist for management of ant-related conjunctival foreign bodies.

Step	Key Actions	Clinical Rationale
Recognition	Identify insect body parts (especially mandibles) embedded in conjunctiva	Prevents misclassification as an inert foreign body
Avoid traction	Do not attempt forceful extraction with forceps alone	Reduces risk of tissue tearing if mandibles are firmly anchored
En bloc removal	Excise conjunctival tissue if mandibles are deeply embedded	Ensures complete removal and prevents retained fragments
Copious irrigation	Perform immediate irrigation; check ocular surface pH if chemical involvement is suspected	Neutralizes possible acid secretion and reduces surface toxicity
Antimicrobial ± steroid	Start broad-spectrum topical antibiotic; add topical steroid if inflammation is significant; taper as appropriate	Prevents infection and controls inflammation
Follow-up	Monitor closely (initially within 1 week, then as clinically indicated)	Detects delayed complications or recurrence of inflammation

Although systemic antibiotics are not routinely required for superficial conjunctival foreign bodies, their use in this case was justified by the depth of conjunctival excision and the potential contamination from insect-related material. Given these risk factors, systemic coverage was selected in addition to topical therapy. Follow-up in this case was limited to one week. While the short-term outcome was favorable, longer follow-up would be necessary to fully exclude late complications such as scarring or recurrence.

## Conclusions

This report describes a rare case of conjunctival injury caused by the Asian weaver ant (*Oecophylla smaragdina*). The case demonstrates that ant-related ocular injuries may necessitate surgical excision rather than simple extraction and should be managed primarily as mechanical trauma, with consideration of possible chemical involvement from formic acid. While this is a single case and generalizability is limited, the observations suggest that prompt irrigation, pH assessment when chemical exposure is suspected, meticulous removal, and appropriate antimicrobial and anti-inflammatory therapy can result in complete recovery with preserved vision. Clinicians should be aware that even small insect foreign bodies may anchor deeply and release irritants, and that careful, stepwise management can prevent vision-threatening outcomes.
